# Human menstrual blood-derived stem cell transplantation suppresses liver injury in DDC-induced chronic cholestasis

**DOI:** 10.1186/s13287-022-02734-1

**Published:** 2022-02-05

**Authors:** Ya Yang, Yanfei Chen, Yalei Zhao, Feiyang Ji, Lingjian Zhang, Shima Tang, Sainan Zhang, Qingqing Hu, Zuhong Li, Fen Zhang, Qian Li, Lanjuan Li

**Affiliations:** State Key Laboratory for Diagnosis and Treatment of Infectious Diseases, National Clinical Research Center for Infectious Diseases, Collaborative Innovation Center for Diagnosis and Treatment of Infectious Diseases, The First Affiliated Hospital, College of Medicine, Zhejiang University, No.79 Qingchun Road, Shangcheng District, Hangzhou, 310003 Zhejiang Province China

**Keywords:** Chronic cholestatic liver injury, Mesenchymal stem cell, Tight junction, Bile transporter

## Abstract

**Background:**

Cholestatic liver injury can lead to serious symptoms and prognoses in the clinic. Currently, an effective medical treatment is not available for cholestatic liver injury. Human menstrual blood-derived stem cells (MenSCs) are considered as an emerging treatment in various diseases. This study aimed to explore the treatment effect of MenSCs in cholestatic liver injury.

**Methods:**

The treatment effect of MenSCs on chronic cholestatic liver injury was verified in 3,5-diethoxycarbonyl-1,4-dihydroxychollidine (DDC)-induced C57/BL6 mice. Pathological, fibrosis area in the liver tissue and serum liver enzymes were tested. Proteomics and western blot were used to explore the related targets and molecular mechanisms. Adeno-associated virus (AAV) 9-infected mice were applied for verification.

**Results:**

MenSCs markedly improved the survival rate of the DDC-treated mice (60% vs. 100%), and decreased the mouse serum aspartate aminotransferase (AST) (169.4 vs. 108.0 U/L, *p* < 0.001), alanine aminotransferase (ALT) (279.0 vs. 228.9 U/L, *p* < 0.01), alkaline phosphatase (ALP) (45.6 vs. 10.6 U/L, *p* < 0.0001), direct bilirubin (DBIL) (108.3 vs. 14.0 μmol/L, *p* < 0.0001) and total bilirubin (TBIL) (179.2 vs. 43.3 μmol/L, *p* < 0.0001) levels as well as intrahepatic cholestasis, bile duct dilation and fibrotic areas (16.12 vs. 6.57%, *p* < 0.05). The results further indicated that MenSCs repaired the DDC-induced liver tight junction (TJ) pathway and bile transporter (OATP2, BSEP and NTCP1) injury, thereby inhibiting COL1A1, α-SMA and TGF-β1 activation by upregulating liver β-catenin expression.

**Conclusions:**

MenSC transplantation could be an effective treatment method for cholestatic liver injury in mice. MenSCs may exhibit therapeutic effects by regulating β-catenin expression.

## Background

Cholestatic liver injury is a rare chronic liver disease characterized by the disruption of bile acid (BA) flow and increased BA concentration in the systemic circulation. Cholestatic liver injury mainly includes primary biliary cirrhosis (PBC) and primary sclerosing cholangitis (PSC) [1, 2]. As the disease progresses, nonspecific symptoms, including pruritus, fatigue, clay stool or bleeding episodes, appear [3, 4]. Long-term continuous cholestasis can develop into liver fibrosis and even cirrhosis and eventually lead to death due to liver failure [5, 6]. A variety of factors, such as drugs, oxidative stress, inflammatory injury and immune disorders, are considered as the causes of cholestasis [7]. Excessive accumulation of bile components, including bile acid, cholesterol and bilirubin, in hepatic and systemic circulation is considered the major driver of liver injury. Currently, adults with different phenotypes of cholestasis have increasingly been evaluated for variants in these genes to identify specific cholestasis-related genes [8, 9].

Treatments for cholestatic liver injury include ursodeoxycholic acid (UDCA) and obeticholic acid (OCA), the only two approved drugs, as well as symptomatic treatments and liver transplantation. Long-term application of UDCA can improve the serum biochemistry and delay the progression of the histological stage. However, approximately 25%–40% of patients do not respond to UDCA [10, 11]. OCA is applied to patients who are insensitive to UDCA, although its administration is associated with several side effects [12, 13]. Additionally, some researchers have indicated that the application of OCA might be associated with an increased risk of liver failure in patients with PBC [14]. PBC and PSC represent major indications for liver transplantation (LT). However, there is a contradiction between the urgent clinical needs and donor liver shortage. In addition, numerous studies have clearly demonstrated that PBC and PSC recur after LT [15]. Therefore, new effective methods for treating cholestatic liver injury are necessary.

Mesenchymal stem cells (MSCs) are novel adult stem cells isolated from various tissues. MSCs can be used in various clinical applications, especially stem cell-based therapies [16]. MenSCs are derived from women’s menstrual blood [17]. Unlike MSCs obtained from other adult tissues such as bone marrow, amniotic fluid, and adipose tissue, MenSCs can be derived through a simple, safe, noninvasive procedure with fewer ethical problems. Compared with MSCs derived from adult bone marrow tissue, MenSCs show a higher proliferation rate [18], indicating that MenSCs may have broader clinical application prospects in the future. Investigators have suggested the effect of MSCs in promoting liver tissue repair and survival rates in acute liver failure, hepatectomy, hepatitis B virus-related acute-on-chronic liver failure, ischemic-type biliary lesions, liver fibrosis, liver transplantation and related graft-versus-host disease [19–26]. Although rash and fever (37–38 °C) that resolved without additional treatment were observed in several patients [27], no MSC transplantation-related safety issues were detected in either short- or long-term follow-up [28, 29]. All these previous studies indicate that MSC transplantation is an ideal candidate for cholestatic treatment. However, the study of the treatment efficacy of MSCs in cholestatic liver injury is limited. It is still unclear whether MSCs can be used as a treatment method and further improve the poor curative effect of drug treatment in patients with cholestasis. To solve the problem, relevant preclinical research and mechanism exploration are necessary.

This study aimed to evaluate the efficacy of MenSC transplantation in mice with DDC-induced cholestatic liver injury. The related molecular mechanism of MenSC treatment in the DDC-treated mice was further elucidated.

## Materials and methods

### Animals

Six- to eight-week-old male C57BL/6 mice were purchased from the Experimental Animal Center of Zhejiang Academy of Medical Sciences and housed under standard conditions.

### Cell culture

MenSCs were cultured in DMEM/F12 (Gibco, Waltham, MA, USA) containing 10% fetal bovine serum (Gibco) and 1% penicillin–streptomycin (Gibco).

### DDC-induced sclerosing cholangitis and MenSC transplantation

C57BL/6 mice were randomly divided into different groups and fed either a control diet (control group) or a diet containing DDC (0.1%).

To evaluate the treatment effect of MenSCs in sclerosing cholangitis, 5 × 10^5^ cells in 500 μL of PBS were injected into mice through the tail vein in the 2nd and 4th weeks of DDC treatment. The mice received MenSC transplantation were considered as the DDC + MenSC group. DDC-fed mice injected with equal amounts of PBS were considered as the DDC group.

After 5 weeks of DDC feeding, mice were anesthetized with 1% pentobarbital sodium and sacrificed. Mouse hepatic tissues were collected immediately. Liver tissues were either fixed in paraffin or stored with frozen liquid nitrogen for further analysis. Peripheral blood was collected and centrifuged to obtain serum separation. The serum samples were stored at − 80 °C.

### Surface markers and differentiation of MenSCs

The expression levels of MenSC surface markers were detected by fluorescence-activated cell sorting (FACS). The collected MenSCs (2 × 10^7^) were washed with staining buffer (Becton Dickinson, Biosciences, San Jose, USA) and incubated for 1 h in diluted antibodies, including CD29, CD34, CD45, CD73, CD90, CD105, CD117 and human leukocyte antigen-DR (HLA-DR) (Becton Dickinson, Franklin Lakes, NJ, USA). Isotype antibodies IgG1 and IgG2a (Becton Dickinson) were applied as negative controls. An FC500 flow cytometer (Beckman Coulter, Pasadena, USA) and FlowJo software (Tree Star, Inc., Ashland, OR, USA) were applied for analysis.

A human mesenchymal stem cell osteogenic differentiation medium kit, chondrogenic differentiation medium kit, and adipogenic differentiation medium kit (Cyagen Biosciences, USA) were used to detect the differentiation potential of MenSCs.

### In vivo tracking of MenSCs

XenoLight DiR (Perkin Elmer, Waltham, MA, USA) was applied to track MenSC migration in vivo. XenoLight DiR solution was diluted and incubated with MenSCs at room temperature for 20 min. Then MenSCs were washed and resuspended in PBS. The resuspended sample was then injected into the control and DDC-treated mice through the tail veins. IVIS analysis (Caliper Life Sciences, Hopkinton, MA, USA) was used after 1 d, 3 d and 7 d.

### Liver function tests

Mouse hepatic function was evaluated by serum aspartate aminotransferase (AST), alanine aminotransferase (ALT), alkaline phosphatase (ALP), direct bilirubin (DBIL) and total bilirubin (TBIL) levels, which were tested using commercial detection kits (Nanjing Jiancheng Bioengineering Institute, Nanjing, China). The measurement of AST, ALT, ALP, DBIL and TBIL was conducted following the instruction of the manufacturer.

### Histological analysis and transmission electron microscopy

For histological analysis, liver tissues fixed in paraffin were embedded in paraffin and sectioned using a MicromHM325 rotary microtome (Thermo Fisher Scientific Life Sciences, USA) for H&E, Sirius Red and Masson staining. The fibrotic area (COL1A1/total area) was quantified using ImageJ software.

For transmission electron microscopy, liver tissues were fixed in 3% glutaraldehyde and rinsed in PBS before being placed in the secondary fixative 1% osmium tetroxide solution for 2 h. Then, the samples were rinsed 3 times in PBS and dehydrated in ethanol by density gradient. The samples were embedded in embedding agent and sectioned at 70 nm thickness. The samples were compared with uranyl acetate and lead citrate, and then examined with transmission electron microscopy (TEM).

### Immunohistochemistry

Paraffin hepatic tissues were used. Samples were deparaffinized and hydrated in ethanol. Hydrogen peroxide (3%) was applied to block endogenous peroxidase. The samples were incubated with COL1A1 (1:1000; Cell Signaling Technology, 72026S) and α-smooth muscle actin (α-SMA) (1:1000; Cell Signaling Technology, 19245) antibodies at 4 °C for 10 h. Then, the samples were washed and incubated with secondary antibody (Abcam, Cambridge, United Kingdom) at 37 °C for 1 h. The samples were stained using 3,3′-diaminobenzidine solution (DAB kit, Abcam) and scanned with a NanoZoomer Digital Pathology system.

### Western blot analysis

Hepatic sample lysates were extracted using RIPA buffer supplemented with cocktail protease and phosphatase inhibitor (Beyotime, Shanghai, China). The BCA protein assay kit (Thermo Fisher Scientific, Rockford, IL) was applied to detect protein concentrations. Equal amounts of total protein were transferred onto polyvinylidene fluoride (PVDF) membranes (Millipore, Bedford, MA, USA) after separation on 4–20% SDS–polyacrylamide electrophoresis gels (GenScript, Nanjing, China). Transferred membranes were blocked in diluted QuickBlock buffer (Beyotime) for 30 min and then incubated at 4 °C for 10 h in the following primary antibodies: β-catenin (1:1000; Cell Signaling Technology, MA, USA, 8480S), Claudin 1 (1:1000; Proteintech Group, Inc., China, 13050-1-AP), Claudin 3 (1:500; Affinity Biosciences, Cincinnati, OH, USA, AF0129), Claudin 5 (1:1000; Affinity Biosciences, AF5216), Claudin 7 (1:1000; Proteintech, 10118-1-AP), NTCP1 (1:1000; Abcam, Cambridge, UK, ab131084), BSEP (1:100; Santa Cruz, sc-74500), OATP2 (1:100; Santa Cruz Biotech, CA, USA, sc-376424), Occludin (1:1000; Cell Signaling Technology, 91131), anti-glyceraldehyde-3-phosphate dehydrogenase (GAPDH) (1:1000; Cell Signaling Technology, 5174). Then, the membranes were incubated in secondary antibodies (1:2000; Cell Signaling Technology) and detected by chemiluminescence reagents (Beyotime). ImageJ software was applied to semiquantify and analyze protein band intensities.

### Protein extraction and digestion

The liver samples were separately treated with liquid nitrogen and suspended in lysis buffer [8 M urea, 50 mM Tris 8.0, 1% NP40, 1% NaDOC (sodium deoxycholate), 5 mM dithiothreitol, 2 mM EDTA, 30 mM nicotinamide, 3 µm trichostatin A, 1% cocktail protease (Sigma, P8215, for use with fungal extracts) and 1% phosphatase inhibitor cocktail (Solarbio, P1260)]. The samples were sonicated and centrifuged at 4 °C for 10 min at 20,000 g to remove tissue fragments. The protein content was detected using a 2D Quant kit (GE Healthcare Life Sciences, PA, USA). Equal amounts of protein were reduced with 5 mM dithiothreitol for 45 min at 30 °C, alkylated with 30 mM iodoacetamide for 1 h in the dark at room temperature, and then precipitated with acetone on ice. The precipitate was washed with acetone and suspended in 0.1 M triethylammonium bicarbonate (TEAB) and digested with trypsin (Promega, Madison, WI) overnight at 37 °C. Digestion was stopped with 1% trifluoroacetic acid. The peptide obtained was cleaned using a Strata X C18 SPE column (Phenomenex, Torrance, CA, USA) and vacuum-dried in a Scan-Vac maxi-beta (Labogene, Alleroed, Denmark).

### TMT labeling and HPLC fractionation

The peptide samples were reconstituted in 120 μL of 0.5 M TEAB and treated using a TMTsixplex label reagent kit (Pierce, Thermo Fisher Scientific, Rockford, IL, 90068). One unit of TMT reagent (5 mg) was thawed and reconstituted in 420 μL of acetonitrile. Subsequently, four reconstituted samples were mixed separately with four TMT reagents (126, 127, 128, and 129), incubated for 2 h at room temperature and then vacuum-dried. Finally, the labeled samples were resuspended in water and mixed. The labeled peptide samples were fractionated for proteome analysis. Fractionation was performed with an XBridge Shield C18 RP column (Waters, Milford, MA, USA) in an LC20AD HPLC system (Shimadzu, Kyoto, Japan).

### Construction and infection with recombinant AAV-Ctnnb1-shRNA

To deplete β-catenin expression in liver tissue, AAV9 that contained Ctnnb1 shRNA (shCtnnb1) and AAV-shControl was constructed by Genomeditech (Shanghai, China). Mice were injected via the tail vein with 150 μL of PBS containing 3 × 10^11^ VG of the AAV-shCtnnb1 or AAV-shControl. At 2 d after virus injection, the mice were fed a DDC or control diet for up to 5 weeks.

### Statistical analysis

All data are reported as the mean ± standard deviation (SD). Student’s *t* test and one-way analysis of variance were used for comparisons of liver function, relative protein expression and fibrotic area among the groups. Graphs were generated using GraphPad Prism 8.0.2 (GraphPad Software, San Diego, CA, USA). *p* values of less than 0.05 were considered statistically significant.

## Results

### Characterization and tracking of MenSCs

**Fig. 1:**
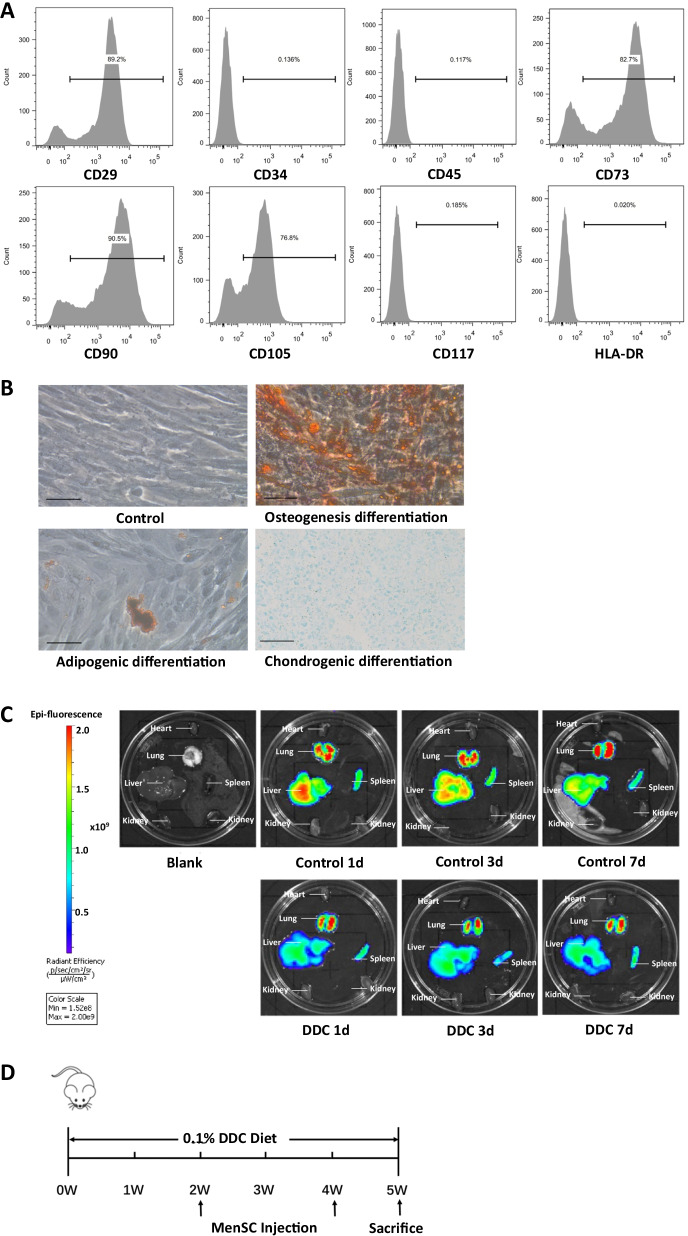
Characterization and tracking of MenSCs. **A** Surface markers of MenSCs were determined using flow cytometer. **B** Cultured MenSCs and differential potential in vitro, Alizarin red staining of osteogenesis differentiation, Oil Red O staining of adipogenic differentiation, Alcian blue staining of chondrogenic differentiation. Scale bar: 50 μm. **C** Analysis of DiR-labeled MenSCs after systemic administration. **D** Schedule of DDC feeding and MenSC transplantation

Flow cytometry was applied to identify the immunophenotype of MenSCs. The results showed positive expression of CD29, CD73, CD90 and CD105 and negative expression of CD34, CD45, CD117 and HLA-DR (Fig. 1A). MenSCs cultured in vitro were spindle-shaped and could be induced to differentiate into osteoblasts, adipocytes, and chondrocytes (Fig. 1B).

To determine the distribution and duration of MenSCs in mice, MenSCs were labeled with XenoLight DiR. Labeled MenSCs were injected into the tail veins of the control and DDC-induced mice, and their distribution was evaluated by in vivo imaging on 1d, 3d and 7d. Fluorescence was detected in lungs, liver and spleen but not in the heart or kidneys (Fig. 1C). The fluorescence could last in vivo for up to 7 days. The schedule of DDC feeding and MenSC transplantation is described above (Fig. 1D).

### MenSCs reduced symptoms caused by DDC in mice

**Fig. 2:**
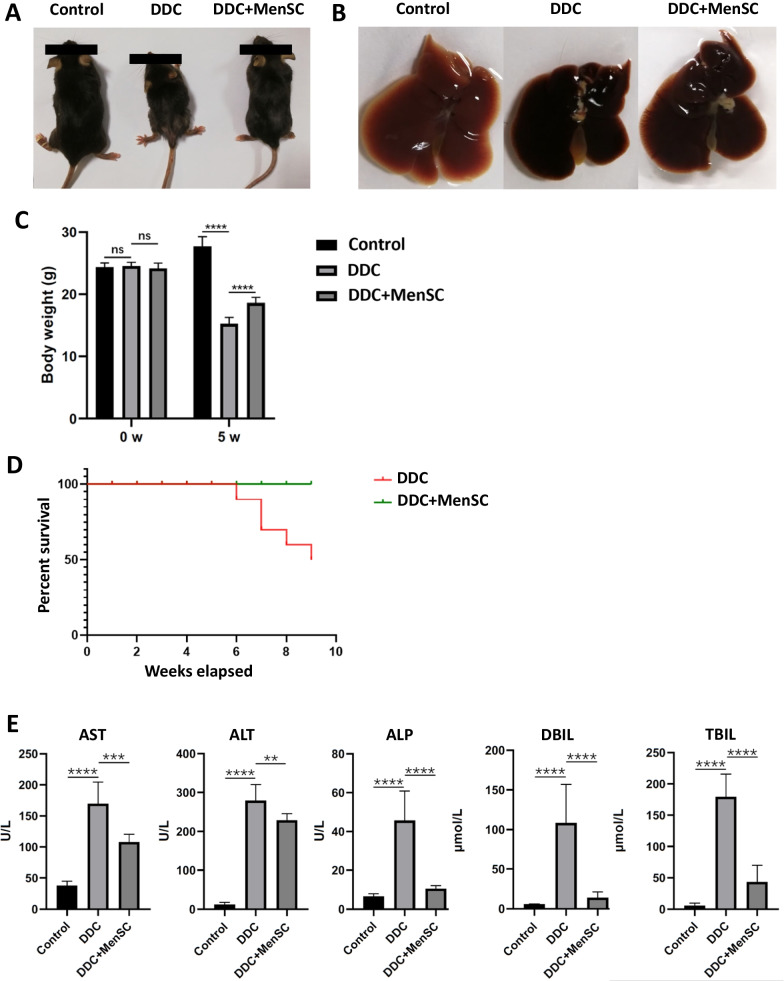
MenSCs reduced symptoms caused by DDC in mice. **A** Representative pictures of the mice in different groups. **B** The livers and gallbladder of mice in different groups. **C** The body weight change of mice in different groups (*n* = 7 for each group). **D** The survival rate of mice at 9 weeks (*n* = 10 for each group). **E** Serum AST, ALT, ALP, DBIL and TBIL levels in different groups (*n* = 7 for each group). **p < 0.01, ***p < 0.001, ****p < 0.0001

Compared with the control mice, the DDC-induced mice showed obvious weight loss, which could be significantly reduced by MenSC transplantation (Fig. 2A, C). Grossly, the livers of the DDC-treated mice were dark red, smaller and stiffer and the gallbladders were enlarged compared with those of the control mice, while MenSC treatment improved these symptoms (Fig. 2B). The survival study showed that 6 mice survived 9 weeks after modeling (6/10, 60%). MenSC transplantation improved the survival rate up to (10/10, 100%) (Fig. 2D). To evaluate liver functions, the AST, ALT, ALP, DBIL and TBIL levels in mouse serum were tested. The results showed that MenSCs could significantly reduce the levels of AST (169.4 vs. 108.0 U/L, *p* < 0.001), ALT (279.0 vs. 228.9 U/L, *p* < 0.01), ALP (45.6 vs. 10.6 U/L, *p* < 0.0001), DBIL (108.3 vs. 14.0 μmol/L, *p* < 0.0001) and TBIL (179.2 vs. 43.3 μmol/L, *p* < 0.0001), indicating that MenSC transplantation could restore DDC-induced mouse liver damage and liver damage-induced jaundice (Fig. 2E).

### MenSC transplantation improved DDC-induced pathological changes in mouse livers

**Fig. 3:**
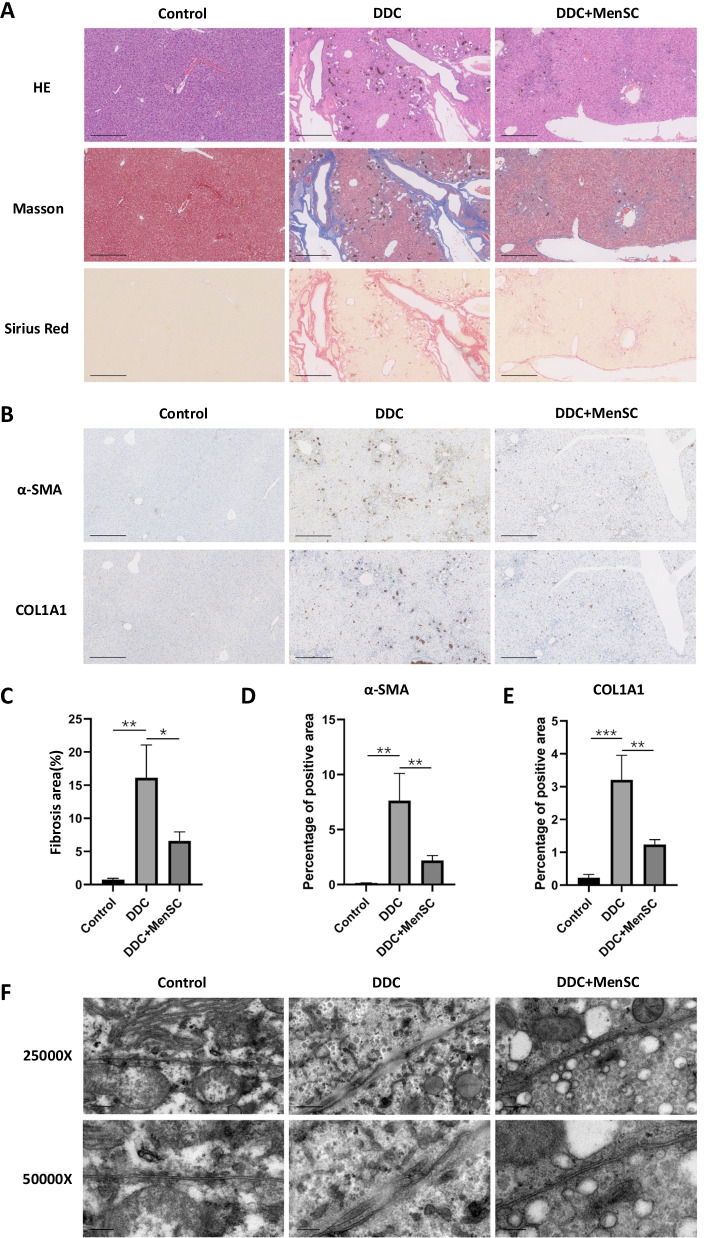
MenSC transplantation improved DDC-induced pathological changes in mouse livers. **A** H&E, Masson and Sirius Red staining of liver sections (*n* = 3 for each group). Scale bar: 250 μm. **B** Representative immunohistochemistry images for α-SMA and COL1A1 (*n* = 3 for each group). Scale bar: 250 μm. **C** Fibrosis area analysis measured from Masson and Sirius Red staining liver sections. **D**, **E** Semi-quantitative analysis of the expression of α-SMA and COL1A1 in liver. **F** Transmission electron microscopy of TJ in different groups. Scale bar: 0.5 μm for 25,000× and 200 nm for 50,000×. *p < 0.05, **p < 0.01, ***p < 0.001

Histological assessment using H&E staining of hepatic tissues revealed periportal ductular reactions, including intrahepatic bile duct dilation and cholestasis, as well as inflammatory cell infiltration in the livers of the DDC group, and these symptoms were significantly relieved in the MenSC group (Fig. 3A). Masson and Sirius Red staining showed fibrotic changes in the hepatic sections of the DDC-treated mice (Fig. 3A). The collagen area percentage in the DDC + MenSC group (6.57%) was significantly reduced compared with the DDC group (16.12%) (*p* < 0.05). Additionally, the expression of α-SMA and COL1A1, markers of hepatic fibrosis, was measured by immunohistochemistry (Fig. 3B). The results indicated that α-SMA and COL1A1 expression was increased in the DDC-treated mice and significantly reduced after MenSC transplantation (*p* < 0.01) (Fig. 3D, E). Transmission electron microscopy of the control liver sections revealed a TJ structure, which is a thin double-stranded electron-dense structure of defined diameter (Fig. 3F). The TJ structure in the DDC livers showed less electron-dense and poorly defined margins and was partly restored in the MenSC group.

### MenSC transplantation altered the liver proteomics profile of the DDC-fed mice

**Fig. 4:**
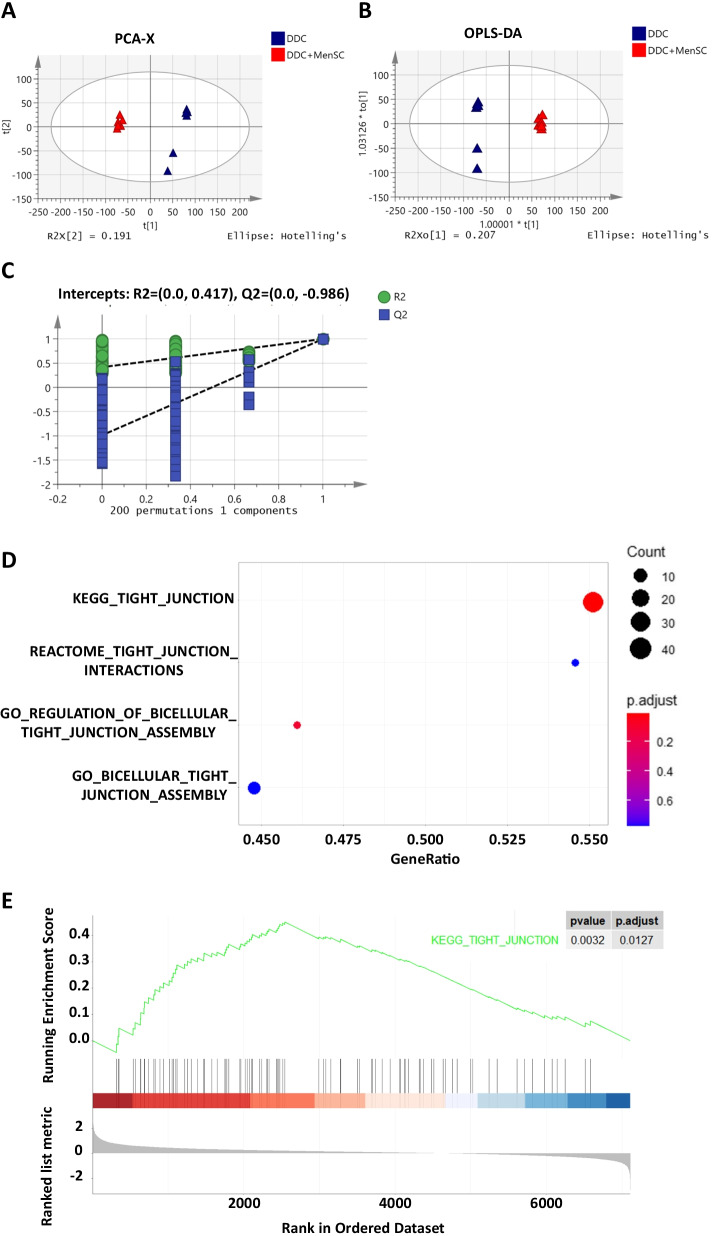
MenSC transplantation altered the liver proteomics profile of the DDC-fed mice. **A** Model of a PCA plot comparing between DDC and DDC + MenSC groups (*n* = 6 for each group). **B** OPLS-DA score scatter plot comparing the DDC and DDC + MenSC groups. Each symbol represents one liver sample (*n* = 6 for each group). **C** Permutation test of the OPLS-DA model. **D**, **E** KEGG, GO and GSEA pathway enrichment analysis

To further explore the molecular mechanism of the treatment efficacy of MenSC transplantation in DDC-induced cholestatic liver injury, a proteomics analysis was performed. Principal component analysis (PCA) and orthogonal partial least-squares-discriminant analysis (OPLS-DA) were used to visualize the proteomic differences in different groups. The PCA score plot revealed no outliers (Fig. 4A). Additionally, no discernible clustering was observed between the DDC and DDC + MenSC groups. The PCA score plot was characterized by the following parameters: R2X = 0.896, Q2 = 0.842. The DDC and DDC + MenSC groups were clearly distinguished in the OPLS-DA score plot (Fig. 4B, C). After analyzing the differentially expressed proteins with Kyoto Encyclopedia of Genes and Genomes (KEGG), Gene Ontology (GO) and Gene Set Enrichment Analysis (GSEA), the TJ signaling pathway attracted our attention (Fig. 4D, E).

### MenSCs promoted TJ- and bile transport function-related protein expression and reduced fibrosis-related protein expression

**Fig. 5:**
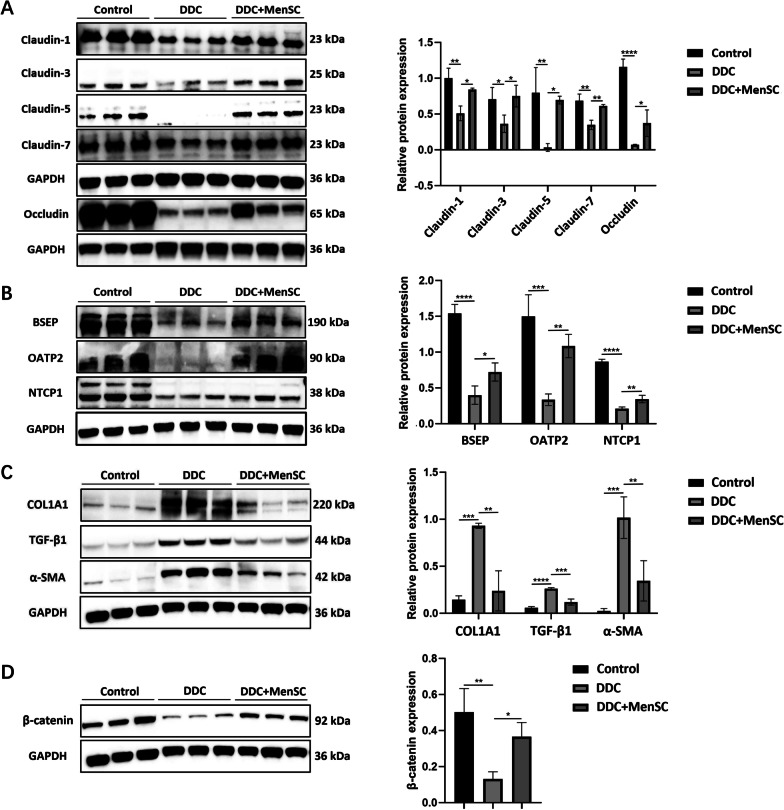
MenSCs promoted TJ- and bile transport function-related protein expression and reduced fibrosis-related protein expression. **A** Western blot analysis protein levels of Claudin-1, Claudin-3, Claudin-5, Claudin-7 and Occludin in liver tissue of different groups (*n* = 3 for each group). **B** BSEP, OATP2 and NTCP1 expression in liver tissues of different groups (*n* = 3 for each group). **C**, **D** Liver COL1A1, α-SMA, TGF-β1 and β-catenin expression among different groups (*n* = 3 for each group). *p < 0.05, **p < 0.01, ***p < 0.001, ****p < 0.0001

To verify our hypothesis, western blot was applied to detect the expression of related proteins. The results indicated that Claudin-1, Claudin-3, Claudin-5, Claudin-7 and Occludin levels were reduced after DDC feeding but restored by MenSC transplantation (Fig. 5A). Considering the significant efficiency of MenSC transplantation in DDC-induced hyperbilirubinemia in mice, the transport function-related proteins BSEP, OATP2 and NTCP1 were also analyzed by western blot. The results indicated that compared with those of the control mice, the BSEP, OATP2 and NTCP1 levels were decreased in the DDC group and restored in the DDC + MenSC group (Fig. 5B). Next, the expression of COL1A1, TGF-β1 and α-SMA, which are proteins related to the fibrosis pathway, was detected. According to the results, COL1A1, TGF-β1 and α-SMA levels were increased in the DDC group but decreased in the DDC + MenSC group (Fig. 5C). It was previously reported that liver-specific β-catenin knockout mice had intrahepatic cholestasis, which resembled DDC-induced symptoms. Thus, the β-catenin levels in liver tissues were measured. β-catenin was significantly inhibited in DDC group and upregulated in DDC + MenSC group (Fig. 5D).

### Liver β-catenin deficiency inhibited the therapeutic efficacy of MenSC transplantation in DDC-induced models

**Fig. 6:**
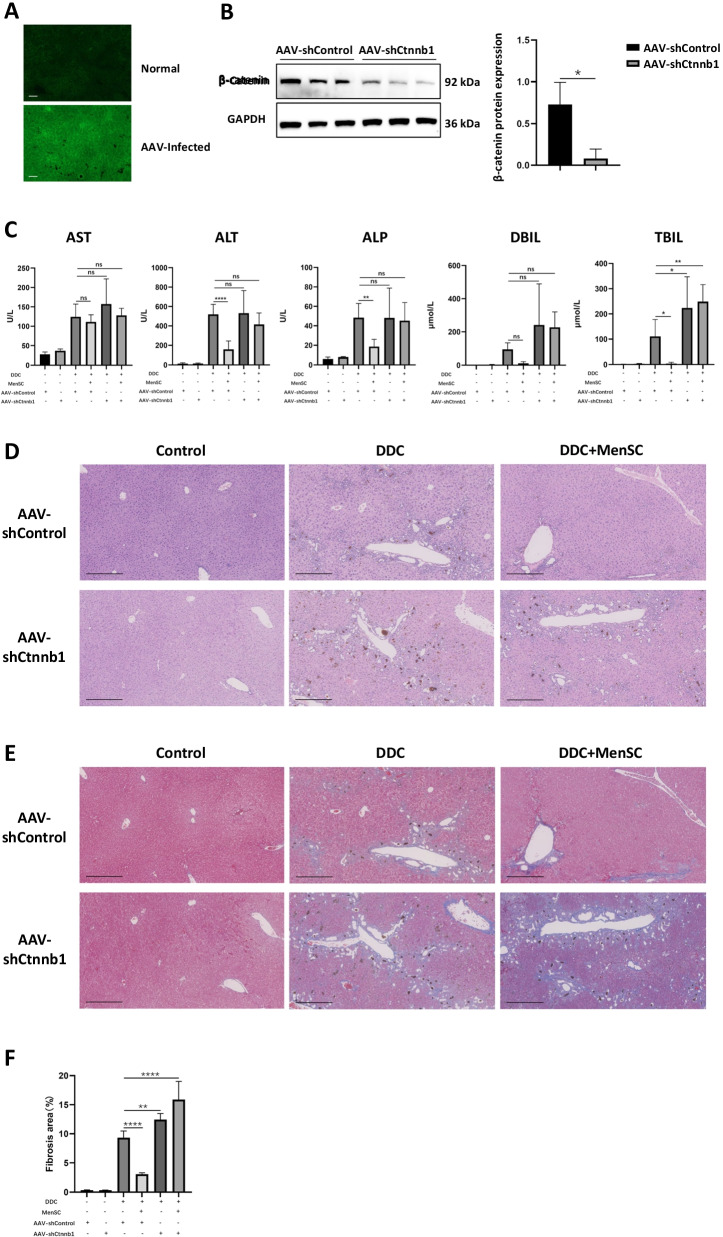
Liver β-catenin deficiency inhibited the therapeutic efficacy of MenSC transplantation in DDC-induced models. **A** Representative images of sections from the control and AAV-infected livers. Scale bar, 50 μm. **B** Expression levels of β-catenin in liver tissues of AAV-shControl and AAV-shCtnnb1 mice (*n* = 3 for each group). **C** Serum AST, ALT, ALP, DBIL and TBIL levels of AAV-shControl and AAV-shCtnnb1 mice in different groups (*n* = 6 for each group). **D**, **E** Representative images of H&E and Masson staining of liver tissues from AAV-shControl and AAV-shCtnnb1 mice in different groups. Scale bar, 250 μm. **F** Fibrosis area analysis (*n* = 5 for each group). *p < 0.05, **p < 0.01, ****p < 0.0001 and n.s. indicates no significance between the two indicated groups

To verify this hypothesis, β-catenin knockdown mice were used. Mice were infected with AAV-shControl or AAV-shCtnnb1. Green fluorescence could be detected in the liver tissues of the AAV-infected mice compared with the liver tissues of normal mice (Fig. 6A). To evaluate the β-catenin knockdown efficiency, the liver β-catenin expression levels were detected by western blot (0.11-fold, *p* < 0.05). Compared with the AAV-shControl mice, the AAV-shCtnnb1 mice showed lower liver β-catenin levels (Fig. 6B). Liver function tests showed that MenSC transplantation failed to downregulate serum AST, ALT, ALP, DBIL and TBIL levels (Fig. 6C). According to the H&E staining of the liver tissues, MenSCs did not significantly improve DDC-induced intrahepatic cholestasis or bile duct dilation in the AAV-shCtnnb1 mice (Fig. 6D). Additionally, MenSC failed to reduce the fibrotic area in the DDC-fed AAV-shCtnnb1 mice (Fig. 6E, F). The results indicated that liver β-catenin deficiency could strongly inhibit the treatment efficacy of MenSC transplantation in DDC-induced liver injury.

### β-Catenin deficiency inhibited the regulatory effect of MenSCs on related proteins

**Fig. 7:**
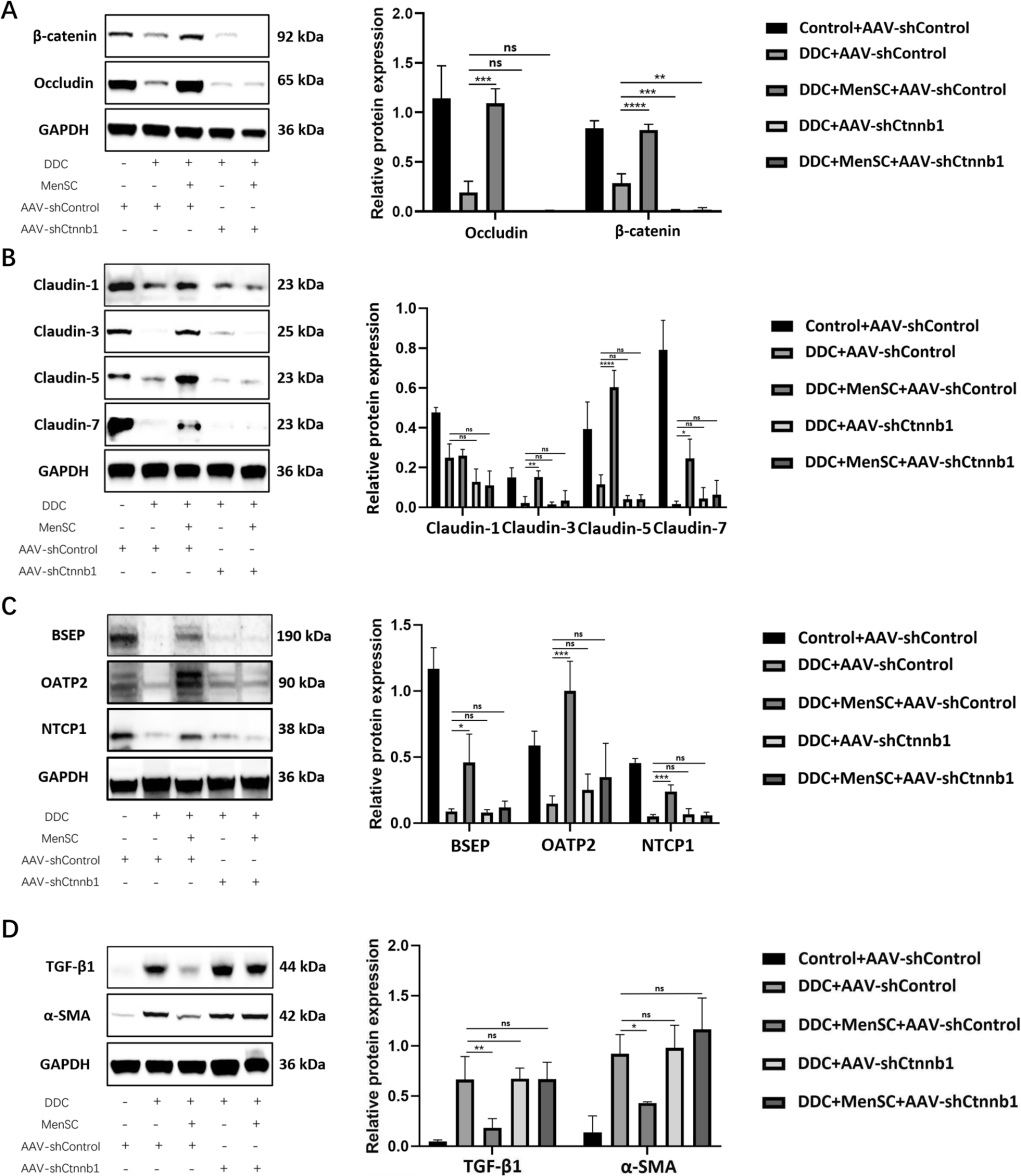
β-Catenin deficiency inhibited the regulatory effect of MenSCs on related proteins. **A** Western blot analysis protein levels of β-catenin and Occludin in the AAV-shControl and AAV-shCtnnb1 infected liver tissues of different groups (*n* = 3 for each group). **B** Expression of Claudin-1, Claudin-3, Claudin-5 and Claudin-7 in AAV-shControl and AAV-shCtnnb1 infected liver tissues (*n* = 3 for each group). **C** Protein levels of BSEP, OATP2 and NTCP1 (*n* = 3 for each group). **D** Expression of TGF-β and α-SMA in liver tissues (*n* = 3 for each group). *p < 0.05, **p < 0.01, ***p < 0.001, ****p < 0.0001 and n.s. indicates no significance between the two indicated groups

To further explore the regulatory effect of β-catenin on related pathways and proteins, western blot was used to assess the expression of proteins. According to the results, MenSC transplantation could not restore DDC-induced damage to β-catenin, Occludin, Claudin-1, Claudin-3, Claudin-5 and Claudin-7 expression in the AAV-shCtnnb1 mice (Fig. 7A, B). Similar results were also found for the BSEP, OATP2 and NTCP1 levels (Fig. 7C). Subsequently, the TGF-β1 and α-SMA expression levels were detected. The results showed that MenSCs failed to inhibit the expression of TGF-β1 and α-SMA in the AAV-shCtnnb1 mice (Fig. 7D). These results indicated that MenSCs regulated the related proteins and pathways by upregulating β-catenin expression.

## Discussion

This study demonstrated that MenSCs could accumulate in the liver and attenuate the development of DDC-induced liver function injury. MenSC therapy reduced intrahepatic bile duct dilation, cholestasis and concomitant fibrosis, which were the main pathological changes in DDC mice. Injected MenSCs significantly reduced DDC-induced mouse mortality. Previous pre-clinical and clinical researches have investigated the potential treatment effect of MSCs in cholestatic liver injury. Fan et al. indicated that UC-MSCs could reduce serum levels of AST, ALT and ALP in 2-octynoic acid coupled to bovine serum albumin-induced PBC mice. Additionally, MSCs are proved to ameliorate liver inflammation by diminishing T cell function [30]. Pinheiro et al. induced cholestatic liver fibrosis by bile duct ligation in mice. According to the results, MSCs conditioned medium injection decreased levels of hepatic enzymes (AST, ALT, ALP and Albumin) and collagen deposition (*p* < 0.001) in liver [31]. In a clinical trial by Wang et al., PBC patients with UDCA resistant received BM-MSC transfusion. After the 12-months follow-up, the serum levels of AST, ALT, γ-GT, DBIL and IgM in PBC patients significantly decreased from baseline. While no significant changes were observed in either TBIL or ALP [32]. These conclusions are consistent with our results. Although previous studies investigated the effect of MSCs in cholestatic liver injury, less studies discussed the mechanisms in depth. Based on this situation, our following research mainly focused on exploring the related mechanisms of MenSC treatment in chronic cholestatic liver injury.

Previously, researchers hypothesized that MSC might exhibit treatment effect in liver injuries by differentiating into hepatocyte-like cells (HLCs) in vivo [33, 34]. However, in recent years, most researchers believe that the MSCs treatment effects are mainly through paracrine activities [35]. According to Chen et al., although transplanted MSCs were recruited to injured liver sites, few cells differentiated into HLCs. MSCs ameliorate hepatic injury via paracrine mediators rather than differentiation effects [18]. Thus, we mainly focused on identifying the target of MenSC treatment in DDC-induced mouse models.

In this study, we found that MenSCs could repair the TJ structure injured in the DDC-treated mice. The blood–bile barrier (BBlB) is primarily composed of TJs [36], and represents a physical barrier formed by liver epithelial cells and hepatocytes, which separates bile from blood sinusoids [37]. Loss of the BBIB is believed to be the main cause of cholestatic liver injury [38]. Although investigators have indicated that MSCs can restore TJ injury in various tissues, such as the colon, hair follicle and brain [39–41], limited researches have demonstrated the TJ repair function of MSCs in the liver. Sato et al. found that liver resident mesenchymal cells could mediate TJ assembly in mouse intrahepatic bile ducts [42]. Our study firstly demonstrated the repair effect of transplanted MSCs on liver TJs. TJ pathway (including Occludin, Claudin-1, Claudin-3, Claudin-5 and Claudin-7) was further proven to be one of the main regulators involved in DDC-induced liver damage and MenSC treatment. Previous studies have proven the regulatory effect of MSCs on related proteins, which confirmed our results. According to Zhang et al., an increase in Occludin and Claudins protein levels could improve liver histology and decrease serum TBIL levels in rats with obstructive jaundice [43]. Pan et al. found that MSC-derived exosomes ameliorated brain ischemic injury by upregulating ZO-1 and Claudin-5 levels [44]. Tak et al. indicated that superoxide dismutase 3-transduced MSCs exhibited protective effect on the epithelial TJ barrier in mouse colitis by preserving the expression of ZO-1 and Occludin [39].

Additionally, the results suggested that MenSCs could restore bile transporter (including OATP2, BSEP and NTCP1) levels inhibited by DDC feeding. Cholestasis could be partly attributed to bile transport function disorder [45, 46]. Various studies have shown that upregulation of OATP2, BSEP and NTCP levels could effectively relieve cholestasis. In the exploration of the treatment effect of UDCA in cholestatic rats, Rost et al. found that UDCA prevented impairment of liver function by restoring bile transporter levels, including Oatp1, Oatp2 and Oatp4 [47]. Xiang et al. suggested that tectorigenin could treat DDC-induced cholestasis by increasing BSEP expression through PPARγ. Blocking upregulation of BSEP expression prevented the therapeutic effect of PPAR on cholestasis [48]. According to Zhang et al., Yin-Zhi-Huang could significantly decrease the serum total bile acids and DBLT levels, and improve histological disorganization by regulating Oatp2, Ntcp, and Mrp2 expression in cholestatic rats [49]. Thus, it was speculated that MenSCs prevent impairment of the liver by restoring the TJ pathway and bile transporter expression.

Liver fibrosis is an important pathological change caused by cholestasis. An early prospective study indicated that more than 50% of patients with stage I-III PBC developed histologically confirmed cirrhosis within 4 years [50]. In the present study, MenSC transplantation reduced liver fibrosis and downregulated TGF-β1 and α-SMA expression in the DDC-treated mice. Pradhan-Sundd et al. suggested that liver fibrosis in cholestatic mice was associated with deregulation of TJs and bile transporters [38]. Although the mechanism of cholestasis leading to liver fibrosis is still unclear, we hypothesized that MenSCs may reduce liver fibrosis by restoring TJ and bile transporter functions.

Furthermore, β-catenin was demonstrated to be a key target of MenSC treatment. MenSCs promote the repair of TJs and bile transport function damage by upregulating the expression of liver β-catenin, which is inhibited by DDC, thereby inhibiting the progression of liver fibrosis. We found that hepatic β-catenin knockdown did not cause significant liver damage in normal mice but inhibited the therapeutic effect of MenSC transplantation in DDC-induced hepatic cholestasis and fibrosis. Furthermore, β-catenin knockdown inhibited MenSC regulation of TJs and bile transport function-related proteins and pathways in the DDC-treated mice.

Thompson et al. reported that the upregulation of β-catenin in mice could enhance the resolution of intrahepatic cholestasis after chronic DDC administration for 150 d [51]. According to Tao et al., mice with β-catenin-deficient hepatocytes demonstrated increased liver injury following the DDC diet [52]. Serum levels of ALT and AST were significantly increased in the KD mice after DDC feeding. These studies are consistent with our results. However, research by Saggi et al. suggested that β-catenin might play an opposite role relative to that in our study in the DDC-induced liver injury model [53]. According to Saggi et al., in mice fed a DDC diet, inhibiting β-catenin could result in decreased liver injury, as evidenced by lower percentage of liver fibrosis area and serum levels of ALP, ALT, AST and TBIL. We hypothesized that β-catenin may play various regulatory roles at DDC-induced liver injury. It means that the regulation of MSCs on β-catenin in cholestasis requires further investigation.

In addition, although it is hypothesized that MenSC transplantation could inhibit liver fibrosis induced by cholestasis, an in-depth study of the mechanism was not conducted. To date, a series of studies have indicated that β-catenin could be a protective factor against cholestasis-induced fibrosis. Interestingly, we observed that β-catenin aggravates hepatic fibrosis in carbon tetrachloride (CCl_4_)-induced models. Li et al. reported that inhibiting the Wnt/β-catenin signaling pathway could attenuate CCl_4_-induced hepatic fibrosis in rats [54]. According to Rao et al., β-catenin promotes hepatic fibrosis by activating hepatic stellate cells in CCl_4_-induced mouse models [55]. The different regulatory effects of β-catenin in different liver fibrosis models attracted our attention. The role of β-catenin in hepatic fibrosis induced by a variety of pathological reasons is worthy of further exploration.

## Conclusion

In summary, the current work investigated the treatment effect of MenSC transplantation in DDC-induced liver injury, and the results suggest that cellular therapy is a promising strategy for cholestatic treatment. Based on the present study, we would further investigate the exact regulatory mechanism of MenSCs on β-catenin in DDC-induced liver injury. Future studies would provide ideas for improving the efficiency of MSCs in cholestasis.

## Data Availability

The mass spectrometry proteomics data have been deposited to the ProteomeXchange Consortium via the PRIDE partner repository with the dataset identifier PXD028425. All data will be made available upon reasonable request to the corresponding authors.

## Abbreviations

MenSCs: Menstrual blood-derived stem cells
DDC: 3,5-Diethoxycarbonyl-1,4-dihydroxychollidine
PBS: Phosphate buffer saline
AAV: Adeno-associated virus
AST: Aspartate aminotransferase
ALT: Alanine aminotransferase
ALP: Alkaline phosphatase
DBIL: Direct bilirubin
TBIL: Total bilirubin
TJ: Tight junction
PBC: Primary biliary cirrhosis
PSC: Primary sclerosing cholangitis
BA: Bile acid
UDCA: Ursodeoxycholic acid
OCA: Obeticholic acid
LT: Liver transplantation
MSCs: Mesenchymal stem cells
HLA-DR: Human leukocyte antigen-DR
α-SMA: α-Smooth muscle actin
GAPDH: Anti-glyceraldehyde-3-phosphate dehydrogenase
TEAB: Triethylammonium bicarbonate
PCA: Principal component analysis
OPLS-DA: Orthogonal partial least-squares-discriminant analysis
KEGG: Kyoto Encyclopedia of Genes and Genomes
GO: Gene Ontology
GSEA: Gene Set Enrichment Analysis
BBIB: Blood–bile barrier
CCl_4_: Carbon tetrachloride
